# Recent Progress in Red Blood Cells-Derived Particles as Novel Bioinspired Drug Delivery Systems: Challenges and Strategies for Clinical Translation

**DOI:** 10.3389/fchem.2022.905256

**Published:** 2022-04-27

**Authors:** Antony Vincy, Sarmistha Mazumder, Amrita, Indranil Banerjee, Kuo Chu Hwang, Raviraj Vankayala

**Affiliations:** ^1^ Department of Biosciences and Bioengineering, Indian Institute of Technology Jodhpur, Karwar, India; ^2^ Interdisciplinary Research Platform Smart Healthcare, Indian Institute of Technology Jodhpur, Karwar, India; ^3^ Department of Chemistry, National Tsing Hua University, Hsinchu, Taiwan

**Keywords:** red blood cells, erythrocytes, nanoerythrosomes, drug delivery systems, nanovesicles

## Abstract

Red Blood Cells (RBCs)-derived particles are an emerging group of novel drug delivery systems. The natural attributes of RBCs make them potential candidates for use as a drug carrier or nanoparticle camouflaging material as they are innately biocompatible. RBCs have been studied for multiple decades in drug delivery applications but their evolution in the clinical arena are considerably slower. They have been garnering attention for the unique capability of conserving their membrane proteins post fabrication that help them to stay non-immunogenic in the biological environment prolonging their circulation time and improving therapeutic efficiency. In this review, we discuss about the synthesis, significance, and various biomedical applications of the above-mentioned classes of engineered RBCs. This article is focused on the current state of clinical translation and the analysis of the hindrances associated with the transition from lab to clinic applications.

## Introduction

**FIGURE 1:**
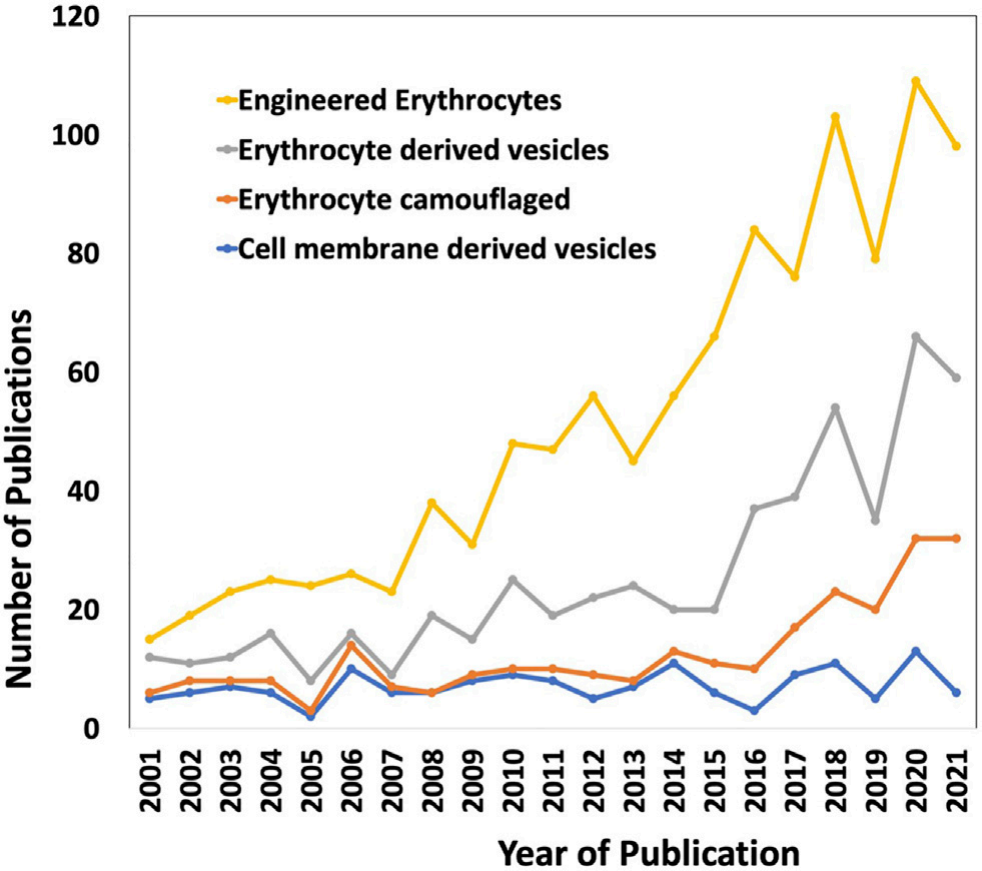
The research trend of using RBCs as delivery vehicles in the past two decades (2001–2021). The keywords used are “Engineered erythrocytes”, “Erythrocyte derived vesicles”, “Erythrocyte camouflaged” and “Cell membrane-derived vesicles”. The data were obtained from Web of Science.

Through the past decades, the evolution of conventional drug formulations to novel drug delivery systems has brought a significant advancement in terms of patient compliance, safety, and efficacy. Researchers have extensively focused on designing optimal drug formulations that have potential to deliver cargos to the desired target sites effectively. Bio-inspired solutions have come into limelight in pursuit of improved circulation, to facilitate release of drugs at the targeted sites, and preventing clearance of drugs from the human system. Erythrocytes/red blood cell (RBC)-derived particles are novel bioinspired drug delivery platforms, in which RBCs are the vital components of the circulatory system and, in combination with different synthetic materials, exhibit the potential to impart both natural benefits and synthetic characteristics for drug delivery. Natural RBCs have a lifespan of 90–120 days in circulation that makes RBCs-derived platforms lucrative for drug delivery applications, as conventional molecular drugs are cleared from the body before exerting adequate therapeutic actions, and produce toxicity when higher dosages of drugs are administered. Apart from delivering a drug to a specific site of interest, the RBC-derived systems also have favorable interactions with the neighboring complex biological environment which stimulates the functional attributes of the drug delivery systems in turn. Owing to the intrinsic biocompatible, biodegradable, and non-immunogenic nature of RBCs, they can be utilized to engineer delivery systems that have the potential for clinical translation. Besides drug loading and physico-chemical conjugation strategies, it is also important to focus on the interactions at the biological interfaces for successful bench to bedside translation (Cai et al., 2018). RBCs are innately the most abundant circulating cells in the biological environment and have inspired the engineering of various man-made RBC-based drug delivery systems in the past several decades. In the last two decades, there is a rapid increase in the research and development of RBC-based drug delivery systems (Figure 1).

**FIGURE 2:**
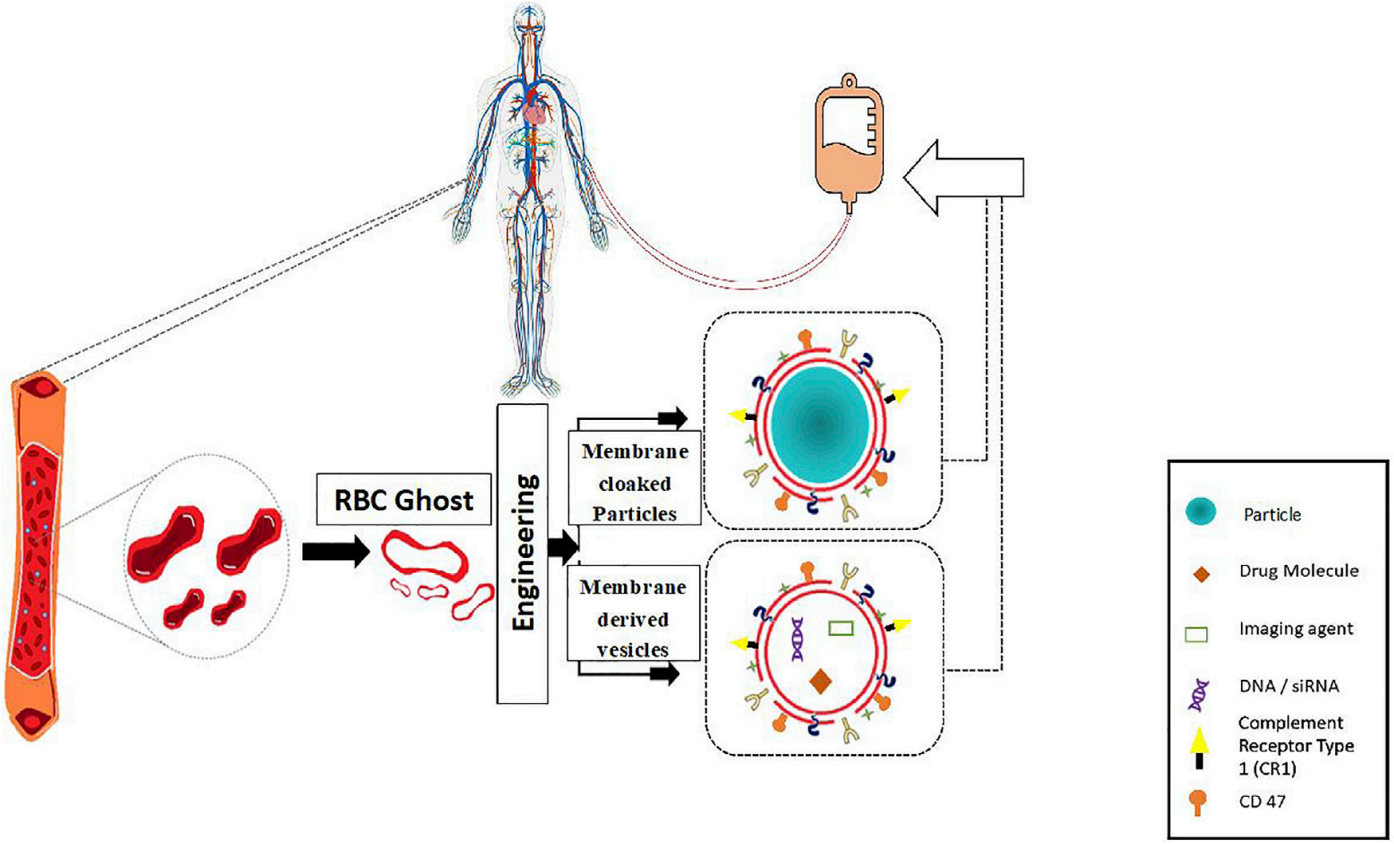
Overview of RBC-based drug delivery systems.

In this review, we summarize the development of RBC coated/camouflaged drug delivery systems along with the popular RBC carrier systems. Various researchers have extensively delved into the exploration and advancement of RBCs as “Smart Delivery Systems” (Han et al., 2018). The last decade has witnessed a vast improvement in drug encapsulation methods for RBC carriers along with the emergence of cell membrane coating technologies for RBC camouflaged delivery systems (Hu et al., 2012; Fang et al., 2018). Engineered RBC carriers have the potential to harbor and transport a vast array of bioactive agents like drugs, imaging agents, enzymes, proteins, and other macromolecules (Hanley et al., 2021). Various strategies have been employed to load RBCs with therapeutic agents without jeopardizing the structural and functional characteristics of RBCs. Additionally, the use of RBC membranes as a camouflaging coating on synthetic carriers like polymeric nanoparticles have been extensively developed in quest for improved circulation half-life and stability. These systems provide combined benefits of a natural coating along with a synthetic core, leading to controlled drug release (Figure 2). RBC-camouflaged drug delivery systems were extensively explored in the field of nanomedicine, and are anticipated to overcome several challenges in the path to clinical translation. RBCs are innately suited for intravascular delivery. Native RBCs are comparatively larger, micron sized cellular components but exploration of their nano dimensions as RBC-based nanocarriers comes with added advantages of drug delivery vehicles. They readily protect the encapsulated cargos and lead to sustained release through their semi-permeable membranes leading to increased therapeutic efficiency. The RBC-coated nanocarriers have prolonged circulation time as compared to bare nanocarriers, combatting the issue of premature clearance in most of the nanocarriers.

This review article is focused on the current state of clinical translation of the RBC based drug delivery systems. We discuss the challenges related to the large-scale fabrication of such constructs, and also revisit the strategies that need to be undertaken to achieve RBC based drug delivery systems for successful translation.

## Synthetic Strategies Involved in the Fabrication of Red Blood Cell-Derived Particles

RBCs are a-nucleated cells, rendering easiness in the method of extraction and purification (Pierigè et al., 2008). The preparation of RBC membrane-derived vesicles and RBC-camouflaged nanoparticles can be categorized into several methods.

### Red Blood Cell Carrier Systems

Encapsulation of therapeutic or imaging agents inside the RBC derived vesicles are mediated through various techniques. Hypotonic treatment is the most commonly used method for synthesizing carrier RBCs without adversely affecting their biochemical integrity (Xia et al., 2019)**.** Following the principle of osmotic lysis, the RBCs swell up and burst when placed in a hypotonic solution, accompanied by the depletion of the intracellular components through pores of sizes ranging from approximately 10–500 nm (Pierigè et al., 2008). There is an entrapment of cargos inside the RBCs by passive diffusion through these pores from the adjacent solution containing drug molecules. The pores on the RBC membrane are reversible and reseal preserving the mechanical integrity and biochemical functions of the membrane. Various other techniques following the same principle of osmotic lysis have also been developed, namely hypotonic hemolysis, hypotonic dilution, hypotonic dialysis and hypotonic pre-swelling (Hu et al., 2012). The hypotonic dilution technique has drawbacks related to low entrapment efficiency and poor circulation life of the resultant construct. To address these drawbacks, hypotonic dialysis was employed and proved to be a suitable method for sustaining physiological and biochemical functionalities of the RBCs.

### Red Blood Cell-Camouflaged Systems

**FIGURE 3:**
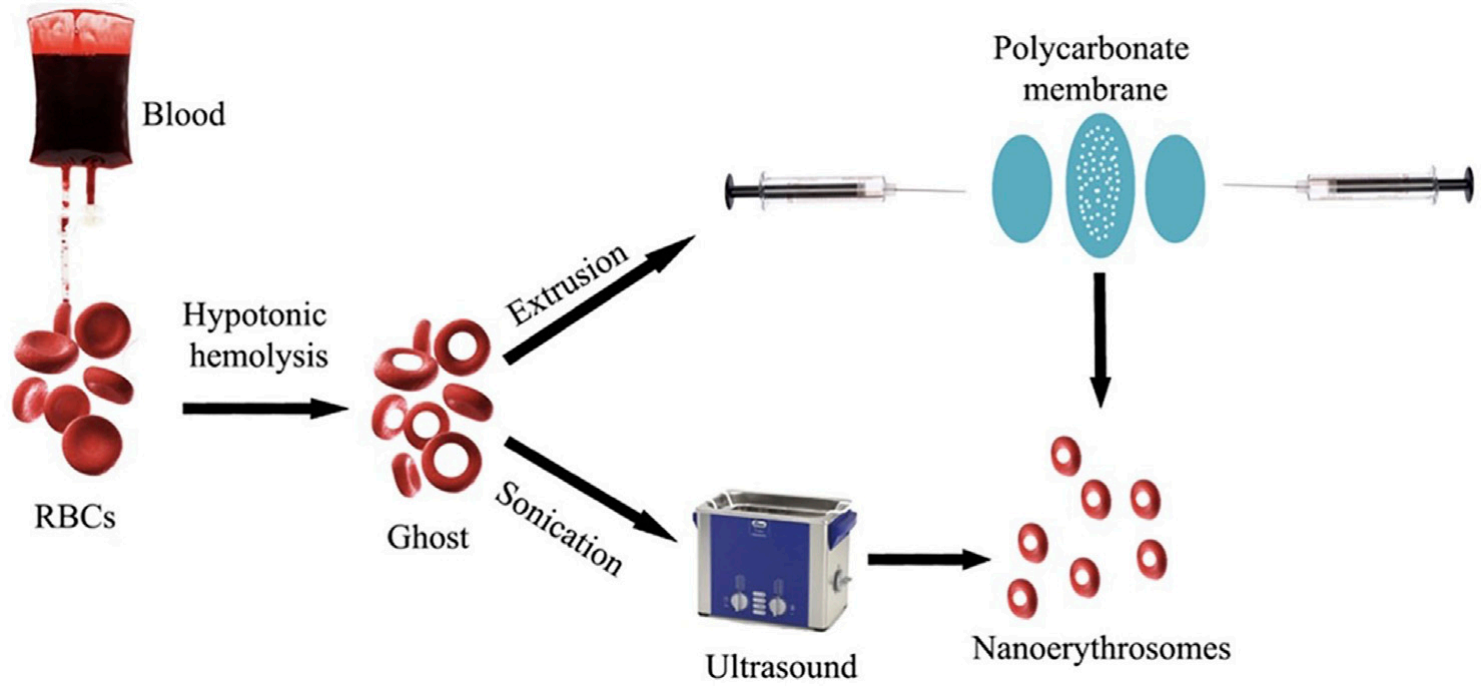
Synthesis of nano-erythrosomes using extrusion and sonication approaches. Adapted from Guido et al. (2021). Copyright @ 2021 (MDPI).

RBC-camouflaged drug delivery systems are an efficient way to coalesce the mechanical and biological advantages of the natural RBCs with those of synthetic materials. The synthesis and assembly of the RBC membranes with the nanoparticles of interest is pivotal for designing RBC-camouflaged nanoparticles. Extrusion (Guo et al., 2018) and sonication are the most commonly used techniques for preparation of RBC-camouflaged nanoparticles (Figure 3) (Guido et al., 2021). However, *in situ* polymerisation and microfluidic electroporation are less popular, and are gradually being explored considering the difficulties in the synthesis processes, reproducibility and scale-up challenges associated with the manufacturing for clinical translation. The biological membrane extrusion method enables achieving uniformly sized particles by passage through porous membranes of required dimensions (Hu et al., 2012; Gao et al., 2013). Extrusion has been reported to be an efficient method to obtain homogenous RBC membrane coating on a poly (D, L-lactic-co-glycolic acid) (PLGA) nanoparticle (HuChe Ming et al., 2011). Other researchers have also demonstrated the utility of the extrusion method to coat RBC membranes on other particles of interest (Piao et al., 2014). However, the major disadvantage of this method is the accumulation of material on the porous membranes that leads to the loss of samples. This has caused hindrance to the applicability of the extrusion technique for large-scale fabrication.

Sonication is another commonly used method to synthesize RBC-camouflaged particles where sound energy is utilised to disperse particles in a liquid sample containing both the RBC membrane and the core particles, using a probe or a bath sonicator. RBC membrane-camouflaged cross-linked 2-hydroxyethyl acrylate (HEA) hydrogel microparticles have been reported to have been synthesised by the sonication method (Hayashi et al., 2018). Sonication is advantageous over extrusion as loss of sample can be avoided during fabrication, but unlike extrusion, sonication may lead to various sizes of the resulting RBC-coated particles. Sonication factors like time, frequency and input power should be cautiously optimised and monitored to reduce chances of protein denaturation. Also, the size and stability of the particles impacted due to sonication makes the search for better synthetic strategies more essential.

Techniques like *in situ* polymerisation and microfluidic electroporation have also been reported to obtain RBC-camouflaged particles. RBC membrane-coated hydrogel nanoparticles have been synthesised through *in situ* polymerisation (Zhang et al., 2015; Zhang et al., 2017a). The RBC membrane-derived vesicles were used as an initiator to form polymeric cores, mediated through radiation or heat. The prospects of this method need to be explored further as it might make absolute coating possible through polymerisation.

**FIGURE 4:**
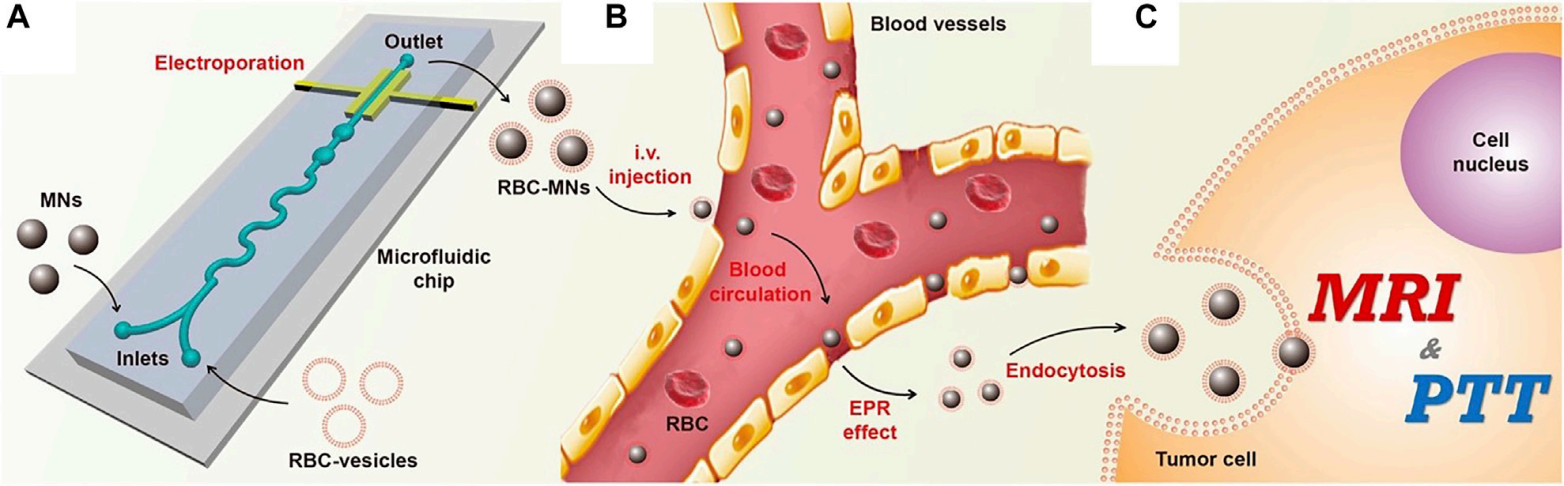
Schematic of microfluidic electroporation-facilitated synthesis of RBC-membrane capped magnetic nanoparticles. **(A)** Microfluidic electroporation facilitates the synthesis of RBC-MNs. **(B)** Subsequently, the RBC-MNs, which are collected from the microfluidic chip, accumulate at the tumor site after the blood circulation. **(C)** Biomimetic RBC-MNs are further used for enhanced *in vivo* tumor MRI and PTT. Adapted from Rao et al. (2017). Copyright @ 2017 (American Chemical Society).

Microfluidic electroporation (Figure 4) enables the formation of pores temporarily in the RBC membrane allowing the passive ingestion of the nanoparticles into the RBC-derived vesicles when exposed to electric impulse. The RBC-camouflaged Fe_3_O_4_ nanoparticles were synthesised using this approach and showed superior colloidal stability, homogenous size distribution and enhanced magnetic resonance imaging (MRI) and photothermal therapy (PTT) *in vivo* as compared to those synthesized using conventional extrusion method (Rao et al., 2017). Microfluidic electroporation technique is expected to address the scale-up and storage challenges faced during industrial translation, and also possess the potential for application in personalised diagnostics and therapeutics as the RBCs can be autologously acquired.

## Characterization of Red Blood Cell-Derived Particles

RBC-derived particles exhibit prolonged circulation time, and can readily evade the phagocytic clearance in the biological environment, due to the retention of RBC membrane proteins and their specialized chemical structure. Besides, the physical characteristics of the RBC-derived particles, the biochemical parameters are also very significant for evaluation of the particle systems. The mechanical characterization of the RBC-derived systems needs further exploration, though. The following criteria are taken into consideration for characterization:

### Physico-Chemical Characterization

Both quantitative and qualitative assessments are crucial for studying the structural integrity of the RBC derived particles. Various techniques have been employed to observe the surface thickness, homogenous distribution, stability, deformability, and permeability of the RBC membrane-derived particles.

Dynamic Light Scattering (DLS) is the commonly employed technique to assess the average hydrodynamic diameter, polydispersity index (PDI) and zeta potential (surface charge) of both the carrier RBCs and RBC-camouflaged particles. There is no significant change in the sizes of carrier RBCs due to cargo loading. But an increase of 10–20 nm can be usually observed in the diameter of the camouflaged nanoparticles as the lipid bilayer of RBC membranes is around 8 nm thick (Su et al., 2016; Zhou et al., 2016). The surface of RBCs is negatively charged due to the presence of ionized carboxyl groups of N-acetyl neuraminic acid (NANA), commonly known as sialic acid. It was reported that the sialic acid was persistent post hypotonic treatment and mechanical extrusion imparting negative charge to the RBC membrane. But sonication presumably results in the degradation of the sialic acid content of the RBC membrane as a decrease in zeta potential of the RBCs and the engineered RBCs was noted in a study (Tang et al., 2022). Hence, the surface charges of both the RBC carriers and RBC-camouflaged nanoparticles have been noted to be negative owing to the negative surface charge of the native RBC membrane.

The shape, morphology, and structural integrity of carrier RBC and RBC-camouflaged nanoparticles are verified by microscopic techniques. Transmission electron microscopy (TEM) and scanning electron microscopy (SEM) offer excellent visualization of the RBC-derived particle systems. The RBC-camouflaged nanoparticles can also be negatively stained with uranyl acetate and observed under TEM to verify their characteristic “core-shell” structure upon successful coating (Wang et al., 2018). Different spectroscopic methods have been employed to study the physicochemical characteristics of the RBC membrane-derived systems. Protein content of the RBC membrane can be quantitatively analyzed by mass spectroscopy technique. UV-visible absorption spectroscopy has been used to confirm successful encapsulation of RBC carrier and RBC camouflaged systems. For RBC-camouflaged systems, Fourier transform infra-red (FT-IR) spectroscopy is an useful method to confirm proper coating (Chugh et al., 2021). Fluorescence co-localization enables introduction of hydrophobic and lipophilic fluorophores into the RBC-camouflaged systems to form “dual-fluorophore labelled” systems, and verifies successful coating and the structural integrity of the “core-shell” arrangement after cellular internalization (Hu et al., 2012).

### Biochemical Characterization

The greatest advantage of RBC-derived drug delivery systems is their ability to achieve a prolonged circulatory effect in the biological environment. Specific RBC membrane proteins present the particles as “self” to the body, thereby decreasing the host immune response and prolonging the systemic circulation. Hence, the verification of surface proteins is of utmost significance to confirm successful coating on the nanoparticles in the RBC-camouflaged systems. Sodium dodecyl sulphate-polyacrylamide gel electrophoresis (SDS-PAGE) and staining with Commassie blue enable the visualization of the proteins to confirm the presence of all proteins on the RBC and the RBC-derived particles, alike (HuChe-Ming et al., 2013). Specific proteins are confirmed with the help of Western blot analysis. CD235a, also known as glycoprotein A (Gagneux and Varki, 1999), is the main RBC sialic acid glycoprotein, and the blood group A antigen is shown to be present in RBC vesicles as well as in RBC-coated nanoparticles. Cluster of differentiation 47 (CD47) is found to be in an equal degree in RBC vesicles, RBC membrane-camouflaged nanoparticles, and RBC cells. Besides analyzing the presence of the surface proteins, their biological activities can also be ascertained through cellular studies. Researchers have demonstrated in mouse macrophage RAW264.7 cell lines that the uptake of RBC-camouflaged nanoparticles was considerably lower than the uptake of naked nanoparticles, thereby indicating the immune escape capability of RBC camouflaged systems. More studies have also been performed to establish the protein orientation and interactions.

### Drug Loading and Release Studies

The drug loading and drug encapsulation efficiencies are the prime factors to dictate the plethora of possible medical applications, which the RBC-based drug delivery systems can achieve. Researchers employ chromatographic techniques to quantify the drugs in the RBC carriers and in the RBC-camouflaged systems. A vast array of biological molecules comprising of nanoparticles, pharmaceutical drugs, imaging agents, and genetic materials, etc. can be encapsulated within the RBC-derived membranes. Drug loading efficiency and drug release behavior have been studied for RBC carriers and RBC-camouflaged particles. It was reported that RBC-camouflaged nanoparticles after 72 h showed only 20% drug release while PEGylated nanoparticles showed 40% drug release, proving that RBC membrane creates a barrier for external diffusion of drugs facilitating gradual and sustained release (Xia et al., 2019). Also, if RBC membrane-derived vesicles contain photosensitizers like hematoporphyrin derivatives, or if RBC-camouflaged nanoparticles contain photosensitive nanoparticles like semiconducting polymer nanoparticles (SPN) (Zheng et al., 2020), then stimulus-responsive, time-dependent drug release studies can be undertaken. Studies have also shown near infrared (NIR) light-initiated and pH-triggered drug release. Drug release studies by many researchers have established that RBC-based drug delivery systems are advantageous in terms of prolonged circulation time and sustained release (Zhang et al., 2021).

## Significance of Engineered RBCs

### Physiology and Mechanical Properties

RBCs are the major components of the circulatory system. These cells are unique as compared to other body cells on account of their long circulation life, high deformability, and lack of organelles. RBCs are 7–8 µm biconcave discs with a high surface area to volume ratio. They survive in circulation for almost 120 days in humans and 50 days in mice without being cleared by macrophages. This property makes RBCs a suitable drug delivery system preventing encapsulated cargos from rapid clearance, thus achieving sustainable release (Han et al., 2018). The extended circulation half-life is a result of the self-markers present on the membrane. CD47 is a surface protein that is highly expressed on the RBC membrane. CD47 specifically binds to signal-regulatory protein alpha (SIRP α) glycoprotein to generate a “do not eat me” signal, that negatively controls the effector functions of innate immune cells including phagocytes (Chambers and Mitragotri 2007). Also, RBC surface proteins like Complement Receptor 1 (CR1) and Decay Accelerating Factor (DAF) can prevent inappropriate self-recognition by an alternate complement pathway affected by C3 Convertase (Fearon 1979). Antibody-mediated complement activation protein referred to as Membrane Attack Complex (MAC) is also inhibited by C8 binding protein (C8bp) present in the RBC membrane, thus escaping immune response. Researchers have reported that CD47 is retained on both micron-sized and nano-sized RBC membranes post mechanical extrusion. Due to the above-mentioned factors, the engineered RBCs are designated as “self” by the human body and subsequently could escape from the phagocytic clearance that any foreign substance in the body is primarily subjected to. Evasion of macrophagic uptake by the RBC carriers and RBC-camouflaged nanoparticles in turn prolongs their circulation time and hence has popularized the concept of RBC-based drug delivery systems. Also, the spleen exhibits a potent filtering mechanism to separate healthy RBCs from the physiologically senescent or pathologically altered RBCs that can be recognized when there is any alteration in size, shape, or deformability. Nano-sized RBC constructs, owing to their lesser diameter, are capable of passing through the splenic endothelial slits and re-entering the circulation.

Healthy RBCs possess appropriate mechanical attributes that differentiate them from aged or damaged RBCs with reduced deformability (Mohanty et al., 2014). Nash et al. have reported an almost 35% increase in the surface viscosity of the membrane of old RBCs in comparison to the young ones (Nash and Meiselman, 1983). Due to a decrease in the deformability, aged RBCs are unable to pass through the endothelial slits of the splenic venous sinus and will be removed from the circulation by phagocytosis in due course. In a similar manner, micron-sized RBC constructs that have been reported to have a decreased deformability remain within the cords unable to pass through the splenic slits, eventually get phagocytosed by the splenic macrophages (Vankayala et al., 2019).

Phosphatidylserine (PS), one of the major phospholipids, usually presents on the inner leaflet of the lipid bilayer of the RBC membrane. During the fabrication of RBC-based particles, there is PS flipping from the inner to the outer leaflet, signaling phagocytic clearance of the particles from the circulation due to macrophage recognition. Researchers have reported a cholesterol enrichment method for the RBC membrane that has successfully reduced PS externalization to enhance the longevity of the RBC-based micron-sized particles in circulation (Tang et al., 2022).

### Toxicity and Immunogenicity

RBC-based drug delivery systems have come into the limelight with a focus on evading phagocytic clearance, leading to prolonged circulation time and less toxicity due to the “self” nature of RBCs in the biological environment. Researchers have studied the circulation characteristics of the RBC membrane-derived vesicles over the synthetic materials, and concluded that retention of CD47 is the major cause to prevent RES uptake and to improve the plasma circulation time (Gao et al., 2013). RBC-derived constructs have been reported to be biocompatible and potentially non-toxic in animal studies. They showed no long-term toxicity in mice where only 20% of the injected particles could be detected after 48 h in the hepatic circulation (Vankayala et al., 2019). Biotoxicity has been evaluated *in vivo* by examining serum biochemistry profiles and histological specimens (Rao et al., 2017). Further short-term and long-term studies are essential to establish the non-toxic behavior of these constructs. RBCs are intrinsically biocompatible, biodegradable and apparently non-immunogenic, yet further evaluation of any other type of immune toxicity, complement activation-related pseudo-allergy (CARPA) or hypersensitivity states need to be conducted. Clarity is required regarding the interaction of the RBC membrane-derived vesicles with the complement system. Researchers have recommended the evaluation of CARPA and hypersensitivity potential of RBC-based delivery systems prior human administration (Malhotra et al., 2022). Utilization of clinically approved components in the RBC-based delivery systems might be advantageous in terms of validating therapeutic potential and immunogenicity. Selection of proper *in vivo* animal models is also crucial for assessment of immune compatibility and systemic toxicity.

## Applications of Red Blood Cell Membrane-Derived Systems

**FIGURE 5:**
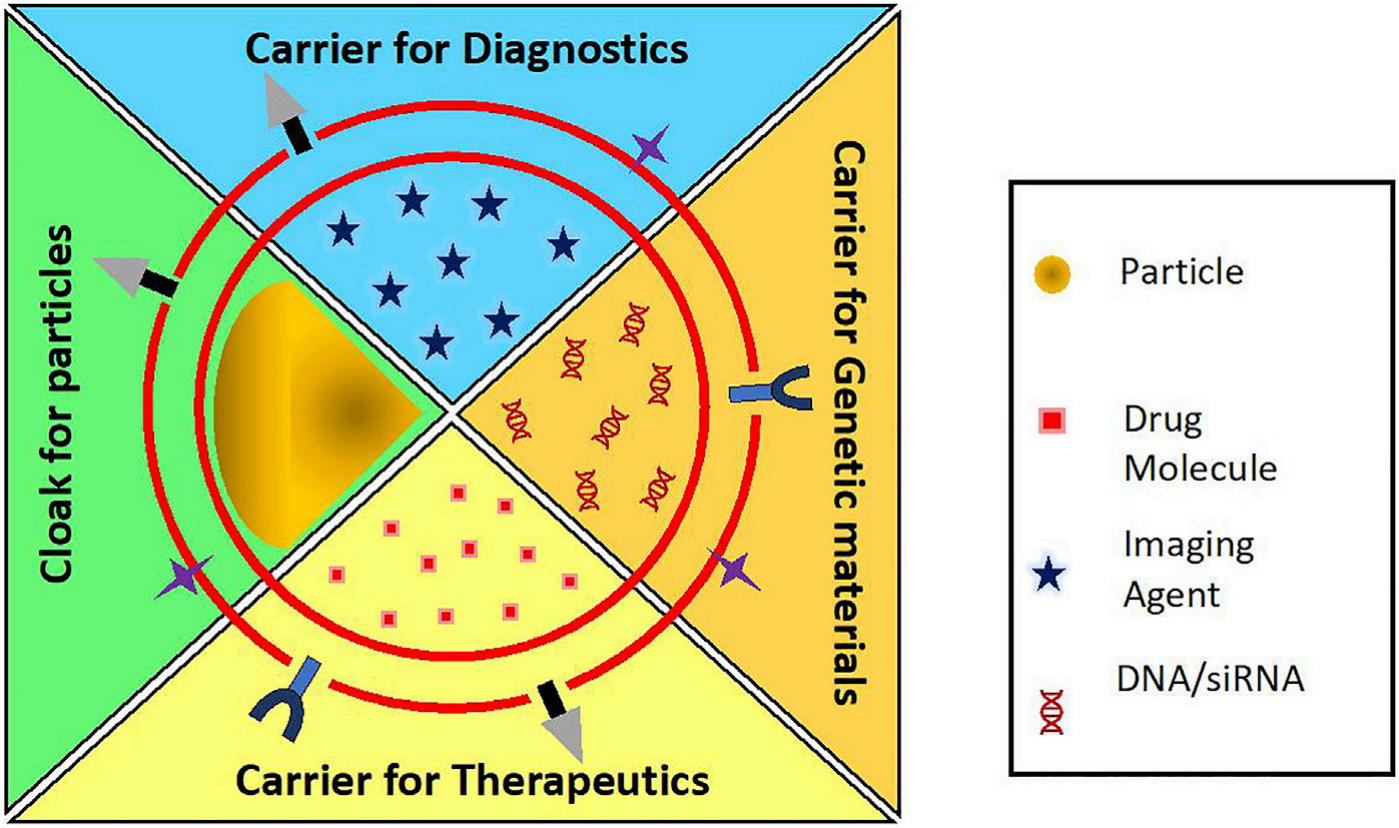
Applications of RBC carriers and RBC-camouflaged systems as carriers for diagnostics, genetic materials, therapeutics, etc.

There has been an expedited development of RBC-based drug delivery systems with the advent of various cargo loading methods and membrane coating techniques. RBC-derived vesicles acting as carrier for drugs and other active agents provide a wide range of opportunities in transporting genes, nanoparticles, drugs, contrast agents that are poorly soluble in water, have low bioavailability and are in need of prolonged circulation period by trapping them which make them more stable and inherently biocompatible. On the other hand, they can also act as camouflaging mantle overlaying the nanoparticles. Some nanomaterials, despite excellent intrinsic properties, cannot be administered in their bare forms because of their immunogenic effects, rapid clearance and toxic bioaccumulation. For example, a very promising photosensitizer, copper sulfide (CuS) nanoplates showed accumulation in spleen, liver, and lungs of mice (Feng et al., 2015). Heavy metal quantum dots like zinc sulfide (ZnS) and cadmium sulfide (CdS) have been shown to Feng et al., 2015 accumulate in zebrafish model (Matos et al., 2020). In order to be used extensively for its outstanding properties, RBC membrane is used as a camouflage to hide the native characteristics of nanoparticles and to endow them with prolonged circulation with minimal or no immune effects and accumulation. Figure 5 shows the applications of the RBC-based drug delivery systems. Table 1 enumerates the various biomedical applications for which RBC-based delivery systems have been explored.

**TABLE 1:** Biomedical applications of RBC-based drug delivery systems.

Material	Load	Loading method	Targeting molecule	Application	Stimuli	Study	References
RBC nano vesicles	Cyanine 5	Co-extrusion	Folic acid	Diagnosis	—	*In vivo*	Chen et al. (2019b)
RBC vesicle	Doxorubicin and chlorin e6	Hypotonic dialysis encapsulation	—	Chemotherapy	Light	*In vitro*	Gao et al. (2017)
RBC coated ultrasmall selenium nano system	Bevacizumab	Extrusion	—	Radiotherapy and antiangiogenic therapy	X-ray	*In vivo*	Liu et al. (2018)
Erythrocyte membrane	Cyclosporine and tacrolimus	Hypotonic dialysis, isotonic resealing and reannealing	—	Immunosuppressive therapy	—	*In vitro*	Biagiotti et al. (2011)
RBC coated iron oxide nanoparticles	Chlorin e6 and doxorubicin	Hypotonic haemolysis	—	Theranostic—image guided combinatorial therapy of PDT and chemotherapy.	Magnetic field	*In vivo*	Wang et al. (2014)
RBC nano vesicles	ICG	Hypotonic haemolysis	—	Theranostic—PTT and fluorescence imaging	Light	*In vitro*	Bahmani, Bacon, and Anvari (2013)
UCNP coated with RBC membrane	Rose bengal and ICG	Hypotonic dialysis	RGD peptide	Theranostic—PDT and fluorescence imaging	Light	*In vivo*	Wang et al. (2019b)
RBC loaded with ferucabuotran	Ferucarbotran	Hypotonic dialysis and isotonic resealing	—	Diagnosis (MRI)	Magnetic field	*In vitro*	Antonelli et al. (2016)
PLGA nanoparticle coated with RBC membrane	Gambogic acid	Extrusion	—	Antiangiogenic therapy	—	*In vivo*	Zhang et al. (2017a)
RBC membranes	Gadolinium based nanoparticles	Hypotonic dialysis and hypertonic resealing	—	Diagnostics	Magnetic field	*In vivo*	Zhang et al. (2016)
Silica nanoparticles coated with RBC membrane	Doxorubin	Hypotonic haemolysis	—	Theranostic—fluorescence imaging and chemotherapy	Light	*In vivo*	Jiang et al. (2018)
RBC coated mesoporous silica	Doxorubicin and chlorin e6	Co-extrusion	Tumour specific ligands	Theranostic—combinatorial PDT and chemotherapy	Light	*In vivo*	Su et al. (2017)
RBC-cancer hybrid membrane coated gold nanorods	Doxorubicin	Extrusion	Homotypic targeting	Theranostic—photoacoustic imaging, combinatorial PTT and chemotherapy	Light	*In vivo*	Li et al. (2020a)
Silver sulfide quantum dots coated with RBC membrane.	Pluronic F-127	Extrusion	—	Theranostic—image guided SDT	Ultrasound	*In vivo*	Li et al. (2020b)
RBC membrane cloaking PLGA nanoparticles	Euphorbiae semen	Extrusion	WSW and NGR peptide ligands	Therapeutic	—	*In vivo*	Cui et al. (2020)

### Cancer

The major disadvantage of conventional chemotherapeutic drugs is the inability to escape from clearance during blood circulation before exerting their therapeutic efficacies. In order to achieve optimal effect, a large dose of drug needs to be administered so that the required amount of drug is able to reach the target site and act effectively. To achieve enhanced bioavailability of drugs in the tumor region even with low concentrations, tumor targeting modifications are required especially for deep-seated tumors. Also, for the diagnostics of deep-seated tumors and circulating/metastatic tumors, conventional imaging system is unable to give properly defined tumor outlines that can be used as reference in surgery. RBC cloaking provide the properties of RBC that makes the diagnostic agent or drug reach any part which makes the drug delivery and diagnostics facile and feasible.

**FIGURE 6:**
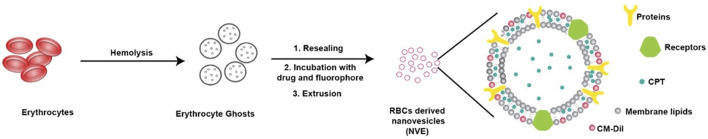
Schematic representation showing usages of RBC membrane for loading a lipophilic drug, camptothecin, for theranostic application. Adapted from Malhotra et al. (2019). Copyright @ 2019 (American Chemical Society).

Camptothecin, a lipophilic drug/dye along with CM-DiI, was co-loaded in RBC-derived nanovesicles for theranostic applications (Figure 6). The nanoparticle was non-phagocytic, and showed strong retention with slow release and superior stealth because of the naturally derived carrier (Malhotra et al., 2019).

**FIGURE 7:**
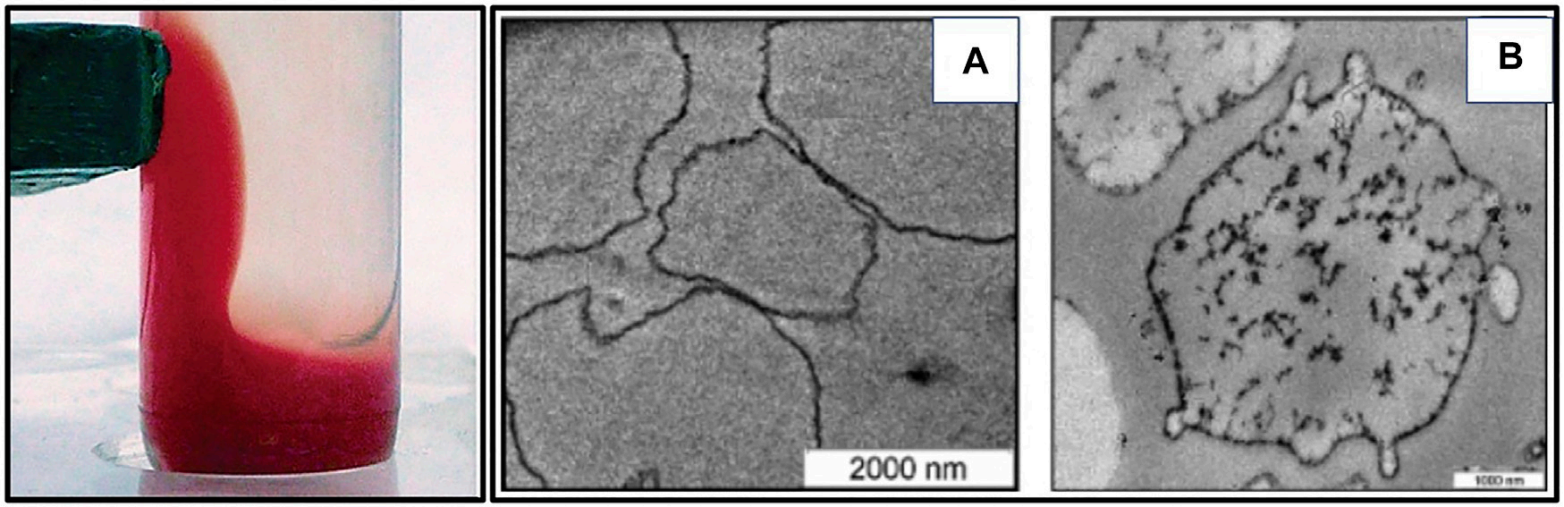
Using RBC membrane as a camouflage for diagnostics. **(A)** Magnetite-loaded RBCs respond to an attached external permanent magnet. **(B)** TEM pictures of **(a)** non-loaded control RBCs and **(b)** SPION-loaded RBCs. Adapted from Brähler et al. (2006). Copyright @ 2006 (American Chemical Society).

To overcome the problems of rapid phagocytosis and short intravascular half-life, contrast agents were cloaked in RBC membranes, thereby circumventing the macrophage system and elimination from the body. Superparamagnetic iron oxide nanoparticles (SPIONs) (Brähler et al., 2006) (Figure 7) have been camouflaged by RBC membrane and reported to show enhanced contrast in MRI for diagnostic applications. Researchers have portrayed several ways of functionalizing contrast agents with RBC membrane for optical imaging and diagnostics applications using nano-erythrosomes (Fornasier et al., 2021).

Combinatorial therapy involving two therapies at the same time is been carried out to achieve better efficacies. Doxorubicin (DOX)-loaded into hollow prussian blue nanoparticle (Chen et al., 2017), paclitaxel and IR-780 co-loaded in lipid multi-chambered nanoparticles (Zhang et al., 2021) and DOX-loaded hollow copper sulfide (CuS) nanoparticles (Wang et al., 2018) were administered for light activated combinatorial therapies of cancer. All these nanoparticles were RBC-cloaked helping them to escape from the reticuloendothelial system (RES) and immune evasion, achieving longer circulation. The drugs or diagnostic agents can also be directly loaded into RBC membranes. A complex of bovine serum albumin (BSA), indocyanine green (ICG) and doxorubicin (DOX) which can be light-activated were encapsulated directly in the RBC vesicles of which the surface was modified with RGD peptide for targeting at the α_v_β_3_-intergrins on tumor membrane accomplishing combinatorial chemotherapy and photothermal therapy. There is also a theranostic nano-riceball with a core made of dextran loaded with DOX and purpurin-18 buried in RBC membrane. The RBC membrane was surface-modified with NGR peptide from CRISPR engineered mice. It is a synergistic nanoparticle capable of responsiveness to endogenous H_2_O_2_ and ultrasound performing sonodynamic therapy (SDT) and chemotherapy under photoacoustic or fluorescence image guidance. This nanoparticle exhibited dramatic inhibition of tumor growths with negligible side effects (Shi et al., 2021).

### Cardiovascular Diseases

For cardiovascular drugs to be impactful, it is demanded to have enhanced stay in the circulation and targeting which could be achieved using nanoparticles (NPs)-based nanocarriers. Though nanoparticles could save the drugs from rapid clearance, it remains a challenge for the nanoparticles to escape from clearance by the reticuloendothelial system (RES) and mononuclear phagocyte system (MPS). The use of RBC-derived membrane to cloak the NPs or RBC-derived vesicles to directly load the drugs makes it a win-win situation to administer any drug on its own and through nanoparticle for the treatment of Cardiovascular Diseases (CVDs). To increase the circulation half-life and to achieve targeting in arthrosclerosis, PLGA nanoparticle loaded with rapamycin was cloaked in RBC derived membrane. This nanocomplex showed superior bio-interface without significant side effects even after long term administration while nanoparticles with polyethylene glycol (PEG) elicited the host immune response after multiple administrations (Zinger et al., 2021).

**FIGURE 8:**
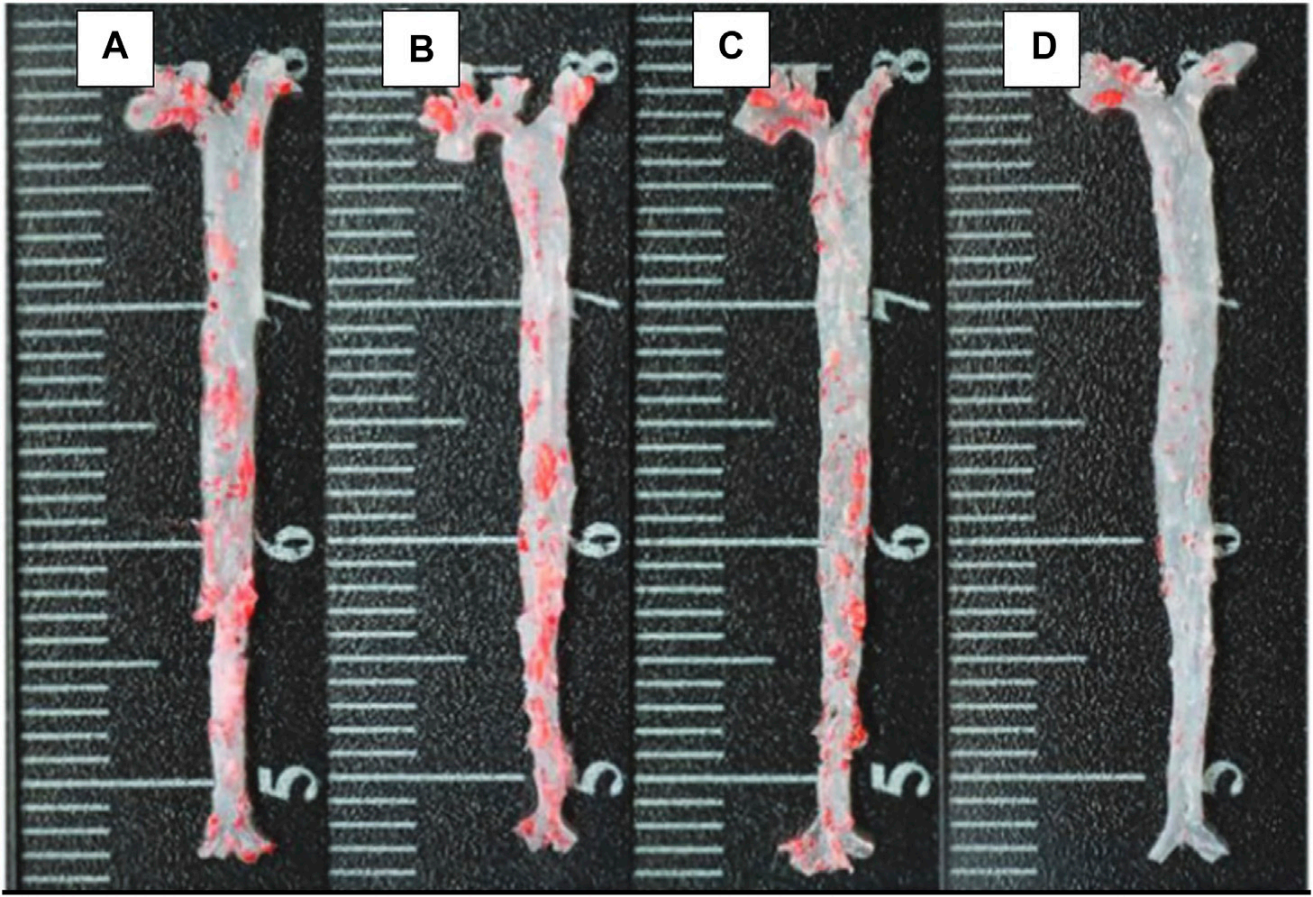
The ORO-stained images of aortas from each group **(A)** control; **(B)** free drug; **(C)** RAP@PLGA; and **(D)** RBC/RAP@PLGA. Adapted from Wang et al. (2019b). Copyright @ 2019 (Wiley).

Comparatively, some researchers reported a delayed progression of arthrosclerosis when the nanoparticle is cloaked with RBC (Figure 8) (Wang P. et al., 2019). For treatment of thrombus, janus nanomotors made of the polymer’s heparin and chitosan, sputter coated with gold, were cloaked with RBC membrane. It was shown that the movement of the motors were controlled by near infrared (NIR) light. Since they were cloaked, they possessed the RBC properties which made their movement more efficient in the relevant biological environments (Shao et al., 2018).

**FIGURE 9:**
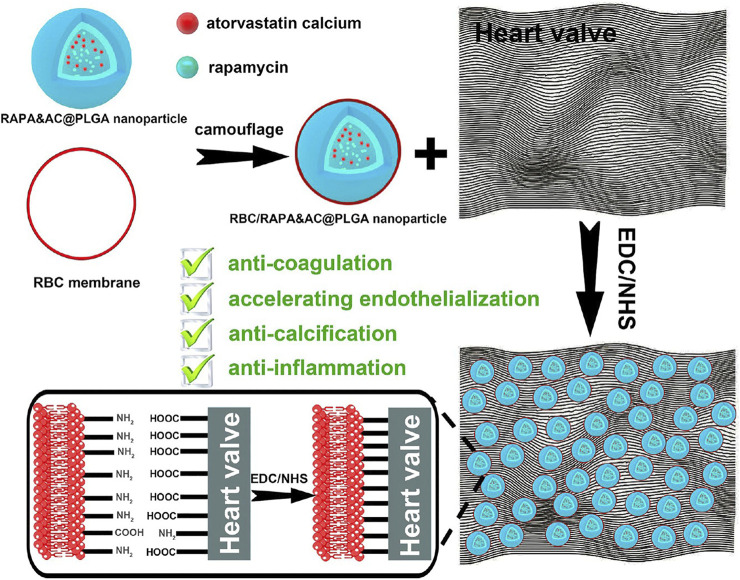
Schematic of erythrocyte membrane biomimetic drug-loaded nanoparticles and mechanism of cross-linking with heart valves for anti-coagulation/accelerating endothelialization process, anti-inflammation and anti-calcification. Adapted from C. Hu et al. (2020). Copyright @ 2020 (American Chemical Society).

Comprehending the inefficacy of fullerenol in thrombolysis *in vivo as* compared to its *in vitro* efficacy, Chen et al. cloaked mesoporous silica nanoparticles bearing the fullerenols with RBC membrane. They claimed that the reduced inefficacy was due to low concentrations of fullerenols at the thrombus site. This intrinsic biotaxi based on cloaking with RBC exhibits an improved the circulation time by 3.1 times, reduced the phagocytosis by 69% and reduced bleeding by 35.3 times (Chen et al., 2020). Considering the thrombosis caused by artificial heart valve, RBC membrane loaded with rapamycin and atorvastatin calcium were cross-linked onto the artificial heart valve. Such a modification improves the biocompatibility with anti-coagulation, anti-inflammation, anti-endothelialization and anti-calcification with the combination of RBC membrane and the drugs it harbors (Figure 9) (Hu et al., 2020).

### Encephalopathy

The shield of the blood brain barrier (BBB) to protect the brain makes it arduous to deliver drugs to the brain. It has been difficult for drug delivery systems to cross the BBB and ingress into the neural region despite considerable advancement in the field. This makes the treatment of brain diseases very challenging and the success rate with regards to encephalopathy stands very low (Li et al., 2021). RBC membrane cloaking will make it trouble-free for any molecule or nanoparticle to have their way across the BBB as their membrane properties possess the privilege of passing through all the systems of the body.

**FIGURE 10:**
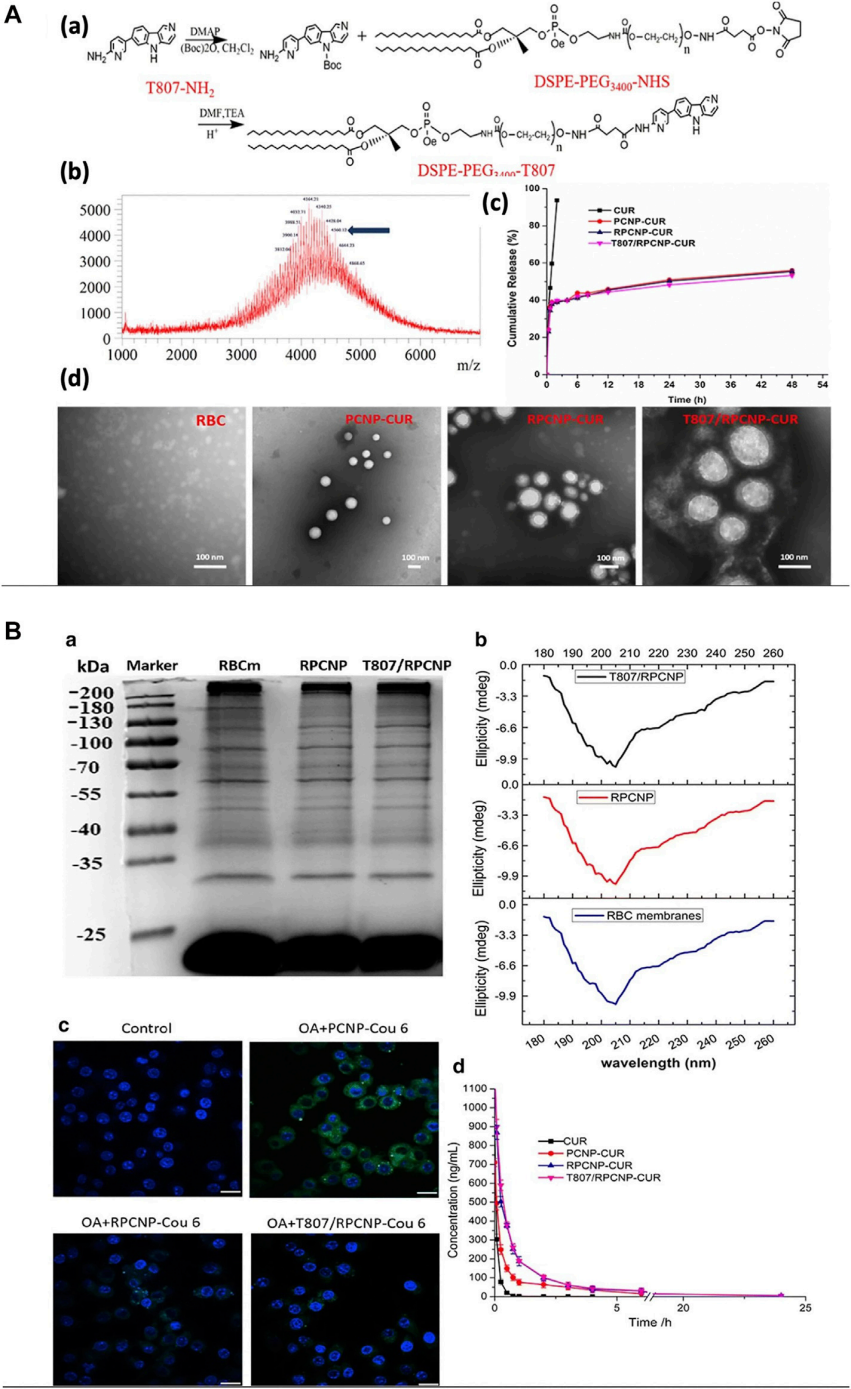
Using RBC membrane as a camouflage for therapeutics. **(A)** Preparation and characterization of nanoparticles. **(B)** The stealth properties of RBC membrane-coated NPs *in vitro* and *in vivo*. Adapted from Gao et al. (2020a).

Curcumin, a multitarget drug, wielded its neuroprotective effects by activating several neurotrophic factors in the brain through various mechanisms. But due to its low water solubility, it has poor bioavailability. The use of curcumin in Alzheimer’s disease to make use of its protective effects was achieved by RBC-cloaked nanoparticles. PLGA nanoparticles loaded with curcumin was cloaked with RBC membrane and functionalized with DSPE-PEG–T807 with a size of 170 nm which could target at neuronal tau. The RBC protects curcumin by increasing its bioavailability while the T807 helps crossing the BBB with good biocompatibility, and sustained curcumin release (Figure 8B) and prolonged circulation (Figure 10) (Gao et al., 2020a). Another study has been designed with the same nanoparticle for Alzheimer’s disease except that human serum albumin was used as a carrier in place of PLGA with a particle size less than 120 nm (Gao et al., 2020b). Both studies have shown good uptakes of the nanoparticles with desired targeting for the treatment of Alzheimer’s. Immunotherapeutic effect in glioblastoma therapy was also achieved by using RBC-cloaked nanogels with a size about 131 nm carrying miRNAs. Reinforcement of microglia and macrophages by miRNAs reprograms the immunotherapeutic impact. The delivery of miRNAs was aided by nanogel cloaked with RBC membrane which contains functional peptides for targeting microglia and macrophage, and also enhanced the fusion with endosomal membrane for increased miRNA release. On a whole, the particle mimicked a virus, delivered and protected miRNAs from degradation with easy travel across the BBB due to the presence of RBC camouflaging (Gao et al., 2021).

### Other Diseases

Most of the drugs, chemicals and molecules used for treatment of multifarious diseases and conditions experience tremendous side-effects. This is because of their poor bioavailability and rapid clearance. RBC cloaking provides refuge by protecting the payloads from endogenous factors, ensuring decrease in concentration fluctuations, longer retention time and reduced dosage (Hamidi et al., 2007).

In a study, primaquine phosphate was used for malaria, but upon broken down metabolites are toxic to blood cells. Delivery of primaquine was stabilized by loading them in RBC membranes which were treated with glutaraldehyde for liver targeting. The drug-loaded erythrocytes have a size of 5.55 µm which were smaller in size than normal RBCs (6.35 µm) (Talwar and Jain 1992). To protect macrophage in murine AIDs, nucleoside analogues were loaded in glutathione-loaded erythrocytes which could deliver the payload drugs selectively to macrophages, thereby reducing the viral load (Fraternale et al., 2002). The same group designed a modified RBC vesicle loaded with glutathione which could be selectively phagocytized by macrophages to exert antiretroviral therapy. RBC as a drug carrier protected the glutathione from being oxidized, and delivered them only in macrophages (Fraternale et al., 2003). Isoniazid, a tuberculosis drug with a rapid clearance rate of 1–1.5 h, was encapsulated in RBC along with magnetite for targeted release under an external magnetic field. Such a controlled release highly reduces the hepatotoxicity and neurotoxicity caused by the drug upon repeated administration (Jain et al., 1997). Fludarabine-loaded RBCs were administered to kill the persistent chronically HIV-1 infected macrophages in circulation, that selectively killed infected macrophages and showed significant efficacy (>98%) in single exposure which lasted for 4 weeks (Magnani et al., 2003). Researchers have also reported RBC coated nano sponges that have shown detoxifying effects in organophosphate poisoning (Chen et al., 2019). The same group has also reported RBC coated nanogels and RBC coated sponges for antibiotic delivery in methicillin resistant *Staphylococcus aureus* (MRSA) infections (Chen et al., 2019a) (Zhang Z. et al., 2017).

## Status of Clinical Translation

RBCs labelled with radiotracers like Tc99m have been successfully used in the clinics as imaging probes for diagnosis of vascular abnormalities. The growing use of radiolabelled RBCs for diagnostics has kindled curiosity and motivation among researchers to explore RBC as a therapeutic agent as well. In pursuit of discovering other avenues for diagnostics and therapeutics, RBC membrane derived vesicles have attracted much interest. Engineered RBCs exhibit great potential to be utilized for various diagnostic and therapeutic purposes, and have slowly but sturdily made it to the clinical development stage from the primary research arena. Despite challenges, varied industrial assistance and government initiatives have enabled the technology to be translated for further clinical and commercial approval (Glassman et al., 2020). Several RBC carrier systems have reached the preclinical or clinical stages but RBC camouflaged systems are yet to overcome the barriers. EryDex, a sustained dexamethasone delivery system, has been developed by an Italian company, EryDel, which showed satisfactory therapeutic efficacy against ataxia-telangiectasia, a rare genetic disease, during its pilot Phase II trial (NCT01255358) (Chessa et al., 2014). The product is under pharmacokinetic investigations in the U.S. and awaiting a Phase III trial at this moment. French pharmaceutical company ERYTECH has expanded the technology by developing a novel solution (GR-ASPA) for treating acute lymphoblastic leukemia in pediatric and adult patients (NCT01518517) (Thomas and le Jeune, 2016). The therapeutic applications of homologous erythrocytes loaded with L-asparaginase (GR-ASPA) have been further supported by a Phase I clinical study in the U.S and other clinical trials in Europe. A trial IIb for investigating the effect of GR-ASPA in acute myeloid leukaemia is in process (NCT01810705) (Agrawal et al., 2013). Therapeutic effects of L-asparaginase encapsulated in RBC combined with Gemcitabine or FOLFOX have also been validated in Phase II trial for progressive metastatic pancreatic carcinoma (NCT02195180) (Hammel et al., 2020). Table 2 lists the details of various RBC-based formulations that have reached different stages of clinical trials.

**TABLE 2:** Overview of clinical trials of various RBC-based formulations (Source: ClinicalTrials.gov).

Technology/company	Disease	Clinical trial phase	Trial identifier	Status
Dexamethasone encapsulated in RBCs/EryDel	Ataxia telangiectasia	Phase 3	NCT03563053	Recruiting Chessa et al. (2014)
L-asparaginase encapsulated in RBCs/ERYtech Pharma	Acute lymphoblastic leukemia	Phase 2	NCT01810705	Completed Thomas and le Jeune. (2016)
—	Pancreatic ductal adenocarcinoma	Phase 3	NCT03665441	Active, not recruiting Hammel et al. (2020)
—	Triple-negative breast cancer	Phase 2/3	NCT03674242	Recruiting
Thymidine phosphorylase encapsulated within autologous erythrocytes/orphan technologies Ltd.	Mitochondrial neurogastrointestinal encephalomyopathy (MNGIE).	Phase 2	NCT03866954	Active, not recruiting
KAN-101/anokion	Celiac disease	Phase 1	NCT04248855	Completed
RTX-134/rubius therapeutics	Phenylketonuria	Phase 1	NCT04110496	Active, not recruiting

Though the RBC-camouflaged systems are unique and superior structurally and functionally than those without RBC-coating layers, there are roadblocks that need to be overcome for a smooth transition from academic settings to clinics. Primarily, there is lack of precise guidelines regarding characterization of these constructs that limit their reproducibility at an industrial scale. Additionally, therapeutic considerations in an actual clinical setting are vital as the *in vitro* and *in vivo* validation studies are restricted to labs and will require stringent modifications. Several factors that are likely to pose as hindrances in the path of clinically translating and commercializing the formulations, need to be catered.

## Summary and Future Perspectives

RBC-based drug delivery systems mimic the natural environment and display robustness, biocompatibility, and adaptability typical of biological systems. As a result, the research community has been exploring RBC-based systems extensively. The arena is still open for various investigations in the field of RBC membrane-derived carriers and RBC-camouflaged nanoparticle systems. Several challenges exist that need to be combatted for smooth transitioning of the RBC-based drug delivery systems from basic research to the clinics that may be enumerated as the following:

• Source of RBC: Laboratory research till date has utilized whole mice or bovine blood or blood from human subjects to fabricate the RBC based constructs. Implementation in the clinics will require blood from individual patients as the technology holds great potential for personalized therapy. Autologous source of blood is the most preferred to validate maximal functionality. These constructs can also be developed using stored blood from the blood bank but possible mechanical alterations and the biochemical fluctuations need to be cautiously explored. RBCs obtained from an erythroid culture system may serve as a source but the process is cumbersome and may not be practically feasible.
• Challenges related to scale-up and reproducibility in batch-to-batch variations: Minuscule differences in the physicochemical characteristics can also cause major pharmacological issues affecting the overall precision of the system. Besides, explicit monitoring of the manufacturing process, standardized protocols for isolation of RBC membranes on a large scale is crucial.
• Post fabrication storage and stability: Use of cryoprotectants is vital for long term storage of the RBC membrane-derived systems. More evidences are essential for selection of correct cryoprotectants and verification of physical and optical stabilities of the constructs in response to the freeze-pump-thaw cycles.
• Patient specific mass manufacturing: The constructs are expected to be sensitive to different blood groups. Donor screening will be indispensable to rule out hemocompatibility issues. Researchers have used freshly obtained blood from human sources or mice blood to fabricate these constructs in the lab but replicating the same in an industrial setting is cumbersome. Utilization of stored blood in this regard can prove advantageous, but studies have reported reduction in membrane protein content, especially a decreased CD47 in stored blood (Kriebardis et al., 2008). CD47 plays a fundamental role in evasion of phagocytic clearance of the RBC-camouflaged nano constructs, hence precise studies need to be conducted to establish the therapeutic potential of the constructs sourced from stored blood.
• Sterilization of RBC-camouflaged delivery systems: Optimal sterilization techniques need to be employed to ensure de-contamination and removal of denatured membrane proteins without compromising the safety and efficacy of the constructs.
• Regulatory approval: Achieving regulatory approvals for nanomedicines has been a tough challenge despite them showing great promises. With extensive ongoing researches globally, these difficulties can be anticipated to be combatted very soon.
• Production economics: Drug development economics is a significant issue that needs to be taken care of meticulously.

The RBC based drug delivery systems have been found to be beneficial in terms of bioavailability, drug loading and controlled drug release. Besides engineering of the delivery systems, major focus should be put on conducting studies, and considering the clinical perspective. Immuno-compatibility of these constructs needs to be strongly established prior to clinical use to minimize cross reactivity. The biodistribution profiles and clearance of these constructs should also be evaluated in larger animal models besides mice. Xenografts from patients can be inspected instead of cell line derived pathologies in mice to achieve analogous results that might serve beneficial for clinical translation. We have also discussed the difficulties in large-scale manufacturing related to commercialization of the RBC delivery systems. The RBC-derived drug delivery systems have great potential to contribute to personalized therapy. Addressing the above-mentioned challenges will bring about a paradigm shift in the field of novel drug delivery systems.
